# Influence of *CCND1* G870A polymorphism on the risk of HBV-related HCC and cyclin D1 splicing variant expression in Chinese population

**DOI:** 10.1007/s13277-015-3401-7

**Published:** 2015-04-08

**Authors:** Zhenzhen Zeng, Jing Tu, Jin Cheng, Mingjie Yao, Yali Wu, Xiangbo Huang, Xiaomeng Xie, Xiaolei Zhang, Fengmin Lu, Xiangmei Chen

**Affiliations:** 1 Department of Microbiology and Infectious Disease Center, School of Basic Medical Science, Peking University Health Science Center, 38 Xueyuan Road, Beijing, 100191 China; 2 Department of Epidemiology and Statistics, College of Public Health, Zhengzhou University, Zhengzhou, China

**Keywords:** Cyclin D1, Polymorphism, Splicing variant, Hepatitis B virus, HCC

## Abstract

The G870A polymorphism in the exon 4/intron 4 boundary of *CCND1* gene is thought to influence the generation of two mRNAs (cyclin D1a and cyclin D1b). The “A” allele codes for a truncated variant, cyclin D1b, which may have higher transforming activity. Herein, the tumor relevance of G870A polymorphism, the association between cyclin D1 variant expression and G870A genotype, and the oncogenic potential of cyclin D1 variants in HBV-related hepatocellular carcinoma (HCC) were examined. We found that there is no significant difference of G870A distribution among the HCC, chronic HBV (CHB) infection, cirrhotic CHB, and healthy control groups. Stratification analysis revealed that in younger patients (ages ≤ 50), cirrhotic CHB patients with AA genotype had an increased risk of developing HCC with odds ratio of 1.943 (95 % CI 1.022–3.694, *p* = 0.0411) as compared with AG/GG genotypes. The two variants were both transcripted from “A” and “G” alleles, and neither cyclin D1a nor D1b production was influenced by G870A genotype in HCC. The expression of both cyclins D1a and D1b decreased in HCC tissues (*p* = 0.003, *p* = 0.005), while increased in adjacent nontumor tissues as compared with normal liver tissues (*p* = 0.045, *p* = 0.034). Overexpression of cyclin D1a or D1b could promote the cell proliferation and cell-cycle progression in Huh-7 and LO2 cell lines. Collectively, our data suggest that G870A polymorphism has only very limited predictive value for HBV-related HCC. Both cyclins D1a and D1b could promote cell proliferation, which might contribute to the potential oncogenic role of cyclin D1 variants during the precancerous cirrhotic stage of hepatocarcinogenesis.

**Electronic supplementary material:**

The online version of this article (doi:10.1007/s13277-015-3401-7) contains supplementary material, which is available to authorized users.

## Introduction

Hepatocellular carcinoma (HCC) is the third cause of cancer-related death worldwide [1] and the 5-year survival rate is only 5 % without treatment [2]. The pathogenesis of HCC is characterized by sequential events including chronic inflammation, hepatocyte hyperplasia, and ultimately malignant transformation [3]. In China, the major risk factor for HCC is the chronic hepatitis B virus (HBV) infection and subsequent hepatic cirrhosis [4]. Although the surgical excision is still the most effective treatment so far, most HCC patients are diagnosed at late stage and cannot be treated surgically. Therefore, identification of more sensitive and specific early biomarkers associated with the increased risk of HCC is crucial for improving HCC management.

Perturbations in the regulation of the core cell-cycle machinery are frequently observed in human cancers. As a key regulator of G1 reentry and progression, cyclin D1 is one of the most frequently altered cell-cycle factors in cancers [5]. Cyclin D1 is encoded by the *CCND1* gene which generates two alternative splice variants (cyclin D1a and cyclin D1b) with different coding sequences. Cyclin D1a is the common isoform containing all five exons, while cyclin D1b derives from retention of intron 4 and contains a premature termination. This structural difference of cyclin D1b renders it to localize in the nucleus through the cell cycle, which may increase its oncogenic potency [6]. G870A polymorphism at the splice donor site of the exon 4/intron 4 boundary, which is thought to affect the production of cyclin D1a and cyclin D1b, was identified as a predictor for increased cancer risk [7–10]. Furthermore, upregulation of cyclin D1b has been observed in several cancers including colorectal cancer, prostate cancer, mantle cell lymphoma, and nonsmall cell lung cancer [11–13], and the change in cyclin D1b/cyclin D1a ratio might lead to unleashed growth of cancer cells [14]. However, contradictory data regarding the polymorphism, D1 variant expression, and correlation with cancer risk have also been reported [15, 16]. Thus, the regulation of alternative splicing of *CCND1* variants might be tissue and race specific.

To date, there is little evidence regarding the role of *CCND1* G870A in HCC susceptibility of patients with chronic HBV infection. In addition, the influence of G870A genotype on the production of cyclin D1 variants and the oncogenic potential of both cyclin D1 variants in HBV-related HCC are not fully understood. In this study, we performed a genotyping analysis in a population-based case–control study with a large cohort, including 238 HBV-related HCC patients, 243 chronic hepatitis B (CHB) patients, 236 cirrhotic CHB patients, and 181 healthy controls. Furthermore, the expression of cyclins D1a and D1b in HCC tissues and the roles of these variants in regulating the cell proliferation were also determined. Our study here provided first evidence that G/A polymorphism is not a strong predictor of the HBV-related HCC risk in Chinese population.

## Materials and methods

### Study population

A total of 717 patients from Youan Hospital in Beijing (October 2009 to December 2011) were enrolled in the case–control study. To avoid the selection bias caused by ethnics, all patients studied were Han Chinese, comprising 243 patients with CHB, 236 patients with cirrhotic CHB, and 238 patients with HCC. The criteria for the diagnosis of CHB group were HBsAg positive, HBV-DNA positive, anti-HBc positive, and the course of disease is more than 6 months, negative for anti-HCV and anti-HIV. Under the premise of compliance with the diagnosis of chronic hepatitis B, the group of cirrhotic CHB patients had persistent or intermittent elevated ALT/AST levels, without evidence of decompensation, the presence of portal venous hypertensive symptoms, such as hypersplenism and mild oesophagogastric varicosity, without variceal bleeding, ascites, or hepatic encephalopathy, and with histological changes of fibrosis F4 by liver biopsy using the Metavir scoring system. HCC patients were all HBsAg positive and diagnosed by ultrasonography and computed tomography and were confirmed by biopsy during liver transplantation or autopsy. In addition, a total of 181 cases of healthy control population were recruited from a health checkup project performed by Beijing Center for Disease Prevention and Control, who did not have a history of liver disease, had no serological evidence of hepatitis B or C virus infection, and without a known history of cancer or genetic diseases.

Another cohort contains 45 pairs of matched primary HCC tumor tissue samples and adjacent nontumor tissue samples which were obtained from patients who underwent routine curative surgery at Henan Tumor Hospital in Zhengzhou, Henan Province of China. All patients were serum HBsAg or HBV DNA positive, and all were histologically diagnosed with liver cirrhosis. None of them has been pretreated with radiotherapy or chemotherapy prior to surgery. Additionally, disease-free liver tissues (*n* = 11) were obtained from liver donors in the same hospital. All these tissues were snap-frozen in liquid nitrogen until examination.

The study protocol was approved by the institute ethics committee, and informed consent was obtained from all patients and donors before the start of study.

### Genomic DNA extraction and *CCND1* G870A genotyping

Genomic DNA was extracted from 2 ml blood sample by using a commercially available DNA extraction kit (Tiangen Genomic DNA Kit; Tiangen Biotech, Beijing, China). All DNA samples were frozen at −20 °C until use. The *CCND1* G870A polymorphism was determined by the Taqman® SNP Genotyping Assay (Life Technologies, Beijing, China) using the 7300 real-time polymerase chain reaction (PCR) system (Applied Bio Systems, USA). About 10 % of samples genotyped with Taqman SNP genotyping assay were further confirmed by the sequencing method.

### RNA isolation and real-time quantitative RT-PCR

Total RNA was extracted from the snap-frozen liver tissues or HCC cell lines with TRIzol reagent (Invitrogen, Carlsbad, CA, USA) and reverse-transcribed with SuperScript® III RT-PCR kits (Invitrogen, Grand Island, NY, USA), according to manufacturer’s protocol. Quantitative RT-PCR was performed to measure the expression levels of cyclin D1a and cyclin D1b using Roche light-cycle 480 sequence detection systems (Roche, Mannheim, Germany), according to the manufacturer’s instructions. Primer sequences used for detection of cyclin D1a were forward: 5′-TGGTGAACAAGCTCAAGTGGAACC-3′ and reverse: 5′-GTGAGGCGGTAGTAGGACAGGAAG-3′. Primer sequences used for detection of cyclin D1b were forward: 5′-AACAGATCATCCGCAAACACGC-3′ and reverse: 5′-GCCTGGGACATCACCCTCACTT-3′. Amplification of the housekeeping gene CTBP1 was performed to normalize the expression levels of cyclin D1a and cyclin D1b. Primer sequences used for CTBP1 were forward: 5′-TTCACCGTCAAGCAGATGAGAC-3′ and reverse: 5′-CTGGCTAAAGCTGAAGGGTTCC-3′.

### Clone sequencing of cyclin D1a and cyclin D1b

The complimentary DNA (cDNA) of human HCC cell lines (Huh-1 and Huh-7) were used for PCR assay by using the specific primers of cyclin D1a or cyclin D1b. Both PCR-amplified fragments for cyclin D1a and cyclin D1b encompassed the exon 4/intron 4 boundary. The primer sequences of cyclin D1a was forward: 5′-CAAACAGATCATCCGCAAACACG -3′ and reverse: 5′-AAGCCTGGTCCACCTCCTCCTC-3′. Primer sequences used for cyclin D1b were forward: 5′-GGAGAACAAACAGATCATCCGCAAAC-3′ and reverse: 5′-GCCTGGGACATCACCCTCACTTAC-3′. After PCR amplification, the PCR products of cyclin D1a or cyclin D1b were purified and cloned into TA vector and transformed into *Escherichia coli Trans-*5α chemically competent cells. Cell clone was randomly picked up and sequenced.

### Western blot assay

Protein was extracted from the snap-frozen liver tissues and cell lines by using the lysis buffer [50 mM HEPES (pH 7.4), 1.5 mM EDTA, 150 mM NaCl, 10 % Glycerol, 10 mM NaF, 1 mM Na_3_VO_4_, 0.5 mM DTT, 1 % Triton X-100, and 1 % Protease Inhibitor Cocktail (P8340, Sigma)]. Protein lysates (30 μg each) were separated by 15 % sodium dodecyl sulfates polyacrylamide gels and transferred onto nitrocellulose membranes (Bio-Rad, USA). After blocking, membranes were incubated with primary antibodies against cyclin D1, Flag tag or tubulin (MBL, Aichi, Japan), overnight at 4 °C and then with the secondary antibodies conjugated with Cy5.5 (Buckinghamshire, UK) for 2 h in room temperature. Finally, the protein–antibody complexes were visualized using the Odyssey Imager (LI-COR Biosciences, Lincoln, NE, USA).

### Cell culture and transfection

The human HCC cell line Huh-7 and human normal hepatic cell line LO2 were maintained in Dulbecco’s modified Eagle medium supplemented with 10 % fetal bovine serum (Gibco, Carlsbad, CA, USA). Cells were transfected with the pFlex vector, pFlex-cyclin D1a, or pFlex-cyclin D1b by using Lipofectamine 2000 (Invitrogen, Carlsbad, CA, USA), according to the manufacturer’s instructions.

### CCK-8 cell proliferation assay

Cells were seeded in 96-well plates at 2 × 10^3^ cells/well. Then every day for a total of 5 days, 10 μl of CCK-8 solution (Dojindo Laboratories, Rockville, MA, USA) was added into each well and incubated for 1 h. The number of cells per well was measured by the absorbance at 450 nm at the indicated time points, according to the manufacturer’s instructions.

### Flow cytometry assay

Flow cytometry assay was used to detect the cell cycle. In brief, cells were washed with phosphate-buffered saline (PBS) twice then resuspended in 2 ml 75 % ethanol and fixed overnight. The fixed cells were resuspended in propidium iodine (PI) solution containing 50 μg/ml RNaseA (Sigma, USA) and incubated at 37 °C for 30 min in the dark. Then the fluorescence of the PI-labeled cells was measured using a flow cytometer (FACSCalibur, BD Biosciences, San Jose, CA, USA).

### Statistical analyses

Hardy–Weinberg equilibrium (HWE) in healthy control population was assessed by the Pearson *χ* ^2^ test. Two-way *χ* ^2^ test and logistic regression analysis were used to compare the differences between CCND1 G870A genotype frequencies and allele frequencies in all groups, and the statistical analysis was accomplished via the Statistical Analysis System (SAS 9.1 TS level 1 M3, Cary, NC, USA). Analysis of variance (ANOVA) test was used to analyze the differences of cyclin D1 expression levels among tumor, nontumor, and normal groups. Linear correlation analysis was carried out to analyze the association between cyclins D1a and D1b messenger RNA (mRNA) expression. The differences of the cyclin D1 protein level in different groups were analyzed by Student’s *t* test. In all cases, a *p* value of less than 0.05 was considered significant.

## Results

### Demography of the study population

The gender distribution among CHB, cirrhotic CHB, HCC and healthy groups showed no differences by the *χ* ^2^ test. However, the age distribution showed a significant increase in HCC patients and cirrhotic CHB patients (53.7 ± 9.4 and 50 ± 9.8 years, respectively), compared to CHB patients and healthy controls (38 ± 13.2 and 34.5 ± 7.9 years, respectively) in our case population. We were unable to obtain a good match in age factor, because the development of liver cirrhosis and tumor is a chronic progressive course, and the average age of the cirrhotic CHB and HCC patients increased with the aggravation of disease.

### Association of G870A polymorphism with HCC risks

The *CCND1* G870A genotype in all subjects was detected by Taqman SNP-genotyping assay, and the association between G870A polymorphism and the risk of HCC were analyzed. First, we performed the test of Hardy–Weinberg equilibrium in our control population. We found that the distribution of G870A genotype in healthy control group was consistent with the Hardy–Weinberg equilibrium, suggesting that there was no population stratification and no sampling bias. Next, we analyzed the correlation between the G870A polymorphism and the susceptibility of HCC in the CHB and cirrhotic CHB groups which all have the background of HBV infection. As shown in Table 1, after adjusted for age and gender by logistic regression analysis, neither the genotype nor allele distribution showed significant difference between HCC and CHB group (*p* = 0.5969 and *p* = 0.5945, respectively) or between HCC group and cirrhotic CHB groups (*p* = 0.1311 and *p* = 0.136, respectively). Similar to the above result, no significant difference of the genotype and allele distribution were observed between HCC and healthy control groups (*p* = 0.7309 and *p* = 0.442, respectively).

**Table 1:** Comparison of *CCND1* G870A genotype and allele frequencies among cases and controls

Groups	AA (%)	AG (%)	GG (%)	*p* Value^a^	OR (95 % CI)^b^	A allele (%)	G allele (%)	*p* Value^a^	OR (95 % CI)
Healthy control	56 (30.9)	91 (50.3)	34 (18.8)	*0.7309*	0.582 [0.222,1.524]	203 (56.08)	159 (43.92)	*0.442*	1.116 [0.848,1.469]
CHB	67 (27.60)	117 (48.1)	59 (24.3)	*0.5969*	1.078 [0.671,1.732]	251 (51.65)	235 (48.35)	*0.5945*	0.934 [0.725,1.202]
Cirrhotic CHB	53 (22.5)	123 (52.1)	60 (25.4)	*0.1311*	0.851 [0.556,1.302]	229 (48.52)	243 (51.48)	*0.136*	0.824 [0.638,1.063]
HCC	67 (28.15)	120 (50.42)	51 (21.43)	*–*	–	254 (53.36)	222 (46.64)	*–*	–

*CHB* chronic hepatitis B virus infection, *HCC* hepatocellular carcinoma

^a^Pearson *χ* ^2^ test

^b^Adjusted for age and gender by logistic regression analysis. GG and GA genotypes were used as reference

We next examined the association between the G870A polymorphism and the HCC risk in the subgroups of the participants based on age, gender, and clinically relevant factors. When subgroup analyses were performed by gender, HBV genotype, serum HBV DNA, ALT, or AST levels, no statistical difference of the G870A distribution was observed among any subgroups of CHB, cirrhotic CHB, and HCC groups (Table S1). However, the stratification analysis by age revealed that the distribution of G870A genotype and allele between younger HCC subgroups and younger cirrhotic CHB subgroups (ages ≤ 50) were slightly different (*p* = 0.0547 and *p* = 0.0572, respectively). Consistent with this result, when compared with the combined 870 AG/GG variant genotypes, cirrhotic CHB patients with the AA genotype had a 1.943-fold odds ratio of developing HCC (95 % CI: [1.022, 3.694], *p* = 0.0411) (Table 2). These findings indicated that the G870A polymorphism was, to some extent, associated with the HCC risk in this subgroup.

**Table 2:** Stratification analysis of cyclin D1 G870A polymorphism and HCC risk in HCC and cirrhotic CHB groups (ages ≤ 50)

Groups	GG + AG	AA	*p* Value^a^	OR (95 % CI)^b^
Cirrhotic CHB	95 (79.8 %)	24 (20.2 %)	***0.0411***	1.943 [1.022,3.694]
HCC	55 (67.1 %)	27 (32.9 %)	*–*	–

^a^Pearson *χ* ^2^ test

^b^Adjusted for age and gender by logistic regression analysis. GG and GA genotypes were used as reference

### Association of G870A genotype with cyclins D1a and D1b expression

**Fig. 1:**
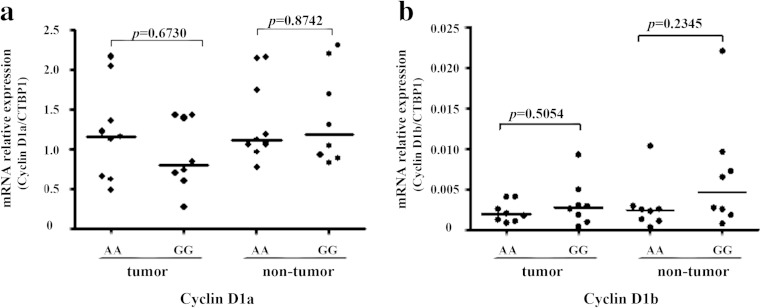
The effect of G870A genotype on the expression of cyclin D1 variants in HCC tissues. The expression of cyclin D1 variants in 17 genotyped HCC tissues was detected by real-time PCR. The expression of cyclin D1a (**a**) or D1b (**b**) in HCC tissues with AA genotype (*n* = 9) was compared to GG genotype (*n* = 8). The housekeeping gene CTBP1 was used to normalize the expression levels of cyclin D1a and cyclin D1b. Each sample was tested in triplicate in two separate experiments

Previous studies have provided evidence that G870A polymorphism may influence the expression levels of cyclin D1a and D1b [17, 18]. To address the contribution of G870A genotype to the production of cyclin D1 isoforms in HCC, we used two independent approaches. Initially, we sequenced the G870A polymorphism in the HCC tissues and compared the expressions of cyclin D1a or cyclin D1b in AA and GG genotype tissues. The results showed that in both HCC tumor and nontumor tissues, the expression of cyclin D1a had no significant difference between AA and GG genotype tissues (*p* = 0.6730 and *p* = 0.8742, respectively; Fig. 1a). Similar results were obtained from cyclin D1b expression (*p* = 0.5054 and *p* = 0.2345, respectively; Fig. 1b). These results suggested that neither cyclin D1a nor D1b production was influenced by G870A genotype in HCC. To support this notion, we then examined the distribution of “A” or “G” alleles in cyclin D1a and cyclin D1b transcripts by clone sequencing analysis in human HCC cell lines Huh-1 and Huh-7, which were identified as AG heterozygous at the 870 locus by genotyping. For this experiment, the fragment of cyclin D1a or cyclin D1b containing 870 locus was amplified from the cDNA of Huh-1 and Huh-7 cell lines by PCR assay with the specific primers of each variant. After PCR amplification, the PCR products were cloned into TA vector and a total of 20 clones of each transcript were randomly picked up for sequencing. If A allele promoted cyclin D1b production, we predicted that the cyclin D1a transcript from AG heterozygous cells should contain more G allele and cyclin D1b transcript should contain more A allele. However, the clone sequencing analysis failed to detect the preference of A allele for cyclin D1b transcript since both cyclin D1a and cyclin D1b transcripts had more G alleles than A alleles in these AG heterozygous cells. Instead, in these two cells, the percentage of clones with G allele was higher than that with A allele in both cyclins D1a and D1b transcripts (Table 3). Taken together, these findings suggested that the two variants of cyclin D1 were both transcripted from A and G alleles, while G allele may have higher transcript efficiency than A allele in liver tissues.

**Table 3:** Clone sequencing analysis of cyclins D1a and D1b in Huh-1 and Huh-7 cells

Cell lines	D1 transcripts (*n* = 20)	A allele	G allele
Huh-1	Cyclin D1a	6 (30 %)	14 (70 %)
	Cyclin D1b	4 (20 %)	16 (80 %)
Huh-7	Cyclin D1a	10 (50 %)	10 (50 %)
	Cyclin D1b	3 (15 %)	17 (85 %)

### Expression of cyclins D1a and D1b in paired HCC tissues

**Fig. 2:**
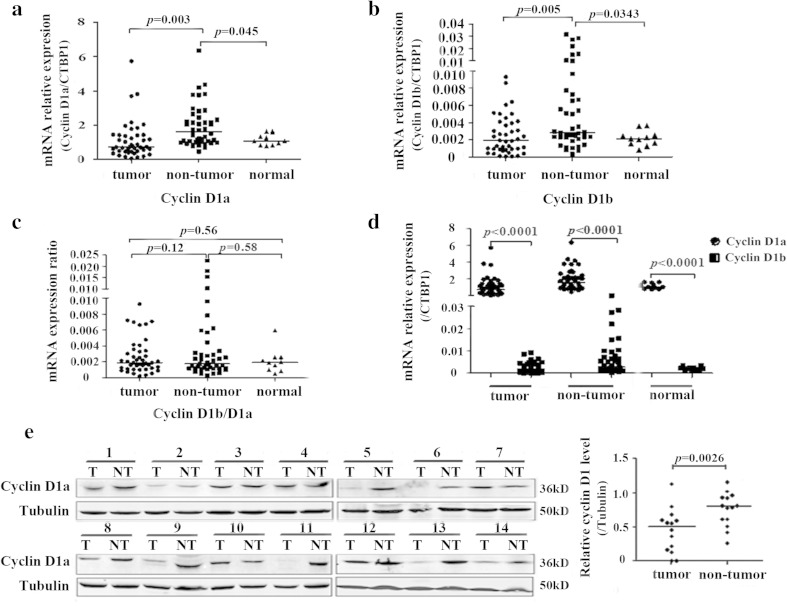
Expression levels of both cyclin D1 variants in paired HCC tissues. Total RNA were prepared from 45 paired HCC tissues and 11 normal liver tissues. The expression of both cyclin D1 variants was quantitated by qRT-PCR and normalized to CTBP1. Each sample was tested in triplicate in two separate experiments. **a** The comparison of cyclin D1a expression levels among the HCC tumor, adjacent nontumor and the normal liver tissues. ANOVA test was used to analyze the difference of cyclin D1a expression levels among these groups. **b** The expression of cyclin D1b was compared as in (**a**). **c** Ratio of cyclin D1b versus cyclin D1a was compared as in (**a**). **d** The difference between cyclin D1a and cyclin D1b expression in the HCC tissues, adjacent nontumor tissues or the normal liver tissues. ANOVA test was used to analyze the difference of cyclin D1 expression levels among these groups. **e** The expression of cyclin D1a protein in 14 paired HCC tissues was detected by Western blot assay. Tubulin was used as an internal loading control in each lane. *T* tumor tissue, *NT* nontumor tissue

Since the cyclin D1b has been shown to have enhanced oncogenic functions when compared with the full-length D1a, we considered whether the expression of cyclin D1b and cyclin D1a or the relative abundance of each variant were dysregulated in HBV-related HCC. We applied a real-time qRT-PCR assay to assess the mRNA expression levels of both variants in an independent cohort of 45 pairs HBV-related HCC tissues and 11 normal liver tissues. As shown in Fig. 2a, the expression level of cyclin D1a was significantly decreased in tumor tissues as compared with the adjacent nontumor cirrhotic tissues (*p* = 0.003). Interestingly, the cyclin D1a expression in the adjacent nontumor cirrhotic tissues was higher than that in normal liver tissues (*p* = 0.045). Likewise, the cyclin D1b expression in tumor tissues was also significantly lower than that in nontumor cirrhotic tissues (*p* = 0.005), which also showed a potential upregulation as compared with normal tissues (*p* = 0.0343; Fig. 2b). We then assessed the relative abundance of each variant by comparing the cyclin D1b/D1a ratio among tumor, nontumor, and normal liver tissues. We found that the cyclin D1b/D1a ratio had no difference in these three tissue groups (Fig. 2c).

Next, we compared the relative expression levels between these two splice variants in the tumor, nontumor, or normal liver tissues after evaluating the efficiency and specificity of both primers (data not shown). We found that the mean mRNA expression level of cyclin D1a was significantly higher than cyclin D1b in tumor tissues (*p* < 0.0001). Likewise, similar results were obtained in nontumor tissues and normal liver tissues (*p* < 0.0001; Fig. 2d). These data suggested that the cyclin D1a variant was the major transcript of *CCND1* gene in liver tissues. To support this notion, we detected the cyclin D1 protein level in 14 pairs of HCC tissues by Western blot assay. Since the cyclin D1 antibody used for Western blot assay is generated from a synthetic peptide corresponding to full length of recombinated cyclin D1a, it can react with both cyclins D1a and D1b. However, we only observed the 36 kDa cyclin D1a in these pairs of HCC tissues and did not observe the 34 kDa cyclin D1b, probably due to the relatively low expression of cyclin D1b in liver tissues. When we compared the cyclin D1a protein level in HCC tumor tissues to their adjacent nontumor cirrhotic tissues, 9 of 14 (64.28 %) tumor tissues showed lower cyclin D1a expression, which is consistent with its lower mRNA level in HCC tumor tissues (Fig. 2e).

### Correlation between cyclins D1a and D1b expression in HCC tissues

**Fig. 3:**
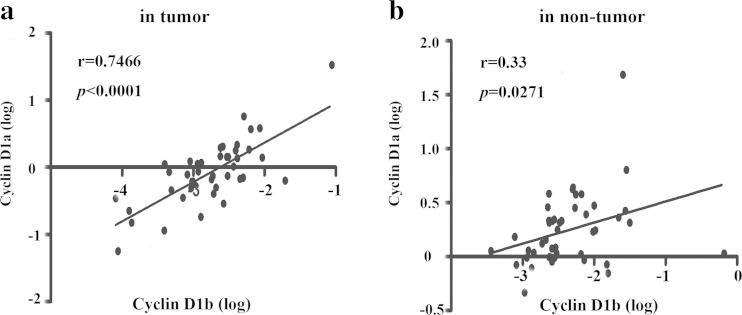
Relationship between cyclin D1a and cyclin D1b expression in HCC tissues. **a** The correlation of cyclin D1a and cyclin D1b expression level in 45 cases tumor tissues was analyzed by liner correction. **b** The correlation of cyclin D1a and cyclin D1b expression level in nontumor tissues was analyzed as in (**a**)

As cyclins D1a and D1b are transcribed from the same gene, it is critical to determine whether the expression of cyclins D1a and D1b is coupled or independent factors in HCC. To address this question, we analyzed the correlation between cyclins D1a and D1b mRNA levels by using Pearson correlations analysis. As shown in Fig. 3a, the level of cyclin D1b had a significant linear correlation with cyclin D1a level in HCC tumor tissues (*r* = 0.7466, *p* < 0.0001). Likewise, the level of cyclin D1b also had a linear correlation with cyclin D1a level in the nontumor tissues (*r* = 0.33, *p* = 0.027), but it is weaker than that in HCC tumor tissues (Fig. 3b). These findings demonstrated that the expression of cyclin D1b might be determined in parallel on the same quantitative platform of cyclin D1a.

### Effect of cyclin D1a or cyclin D1b on cell proliferation

**Fig. 4:**
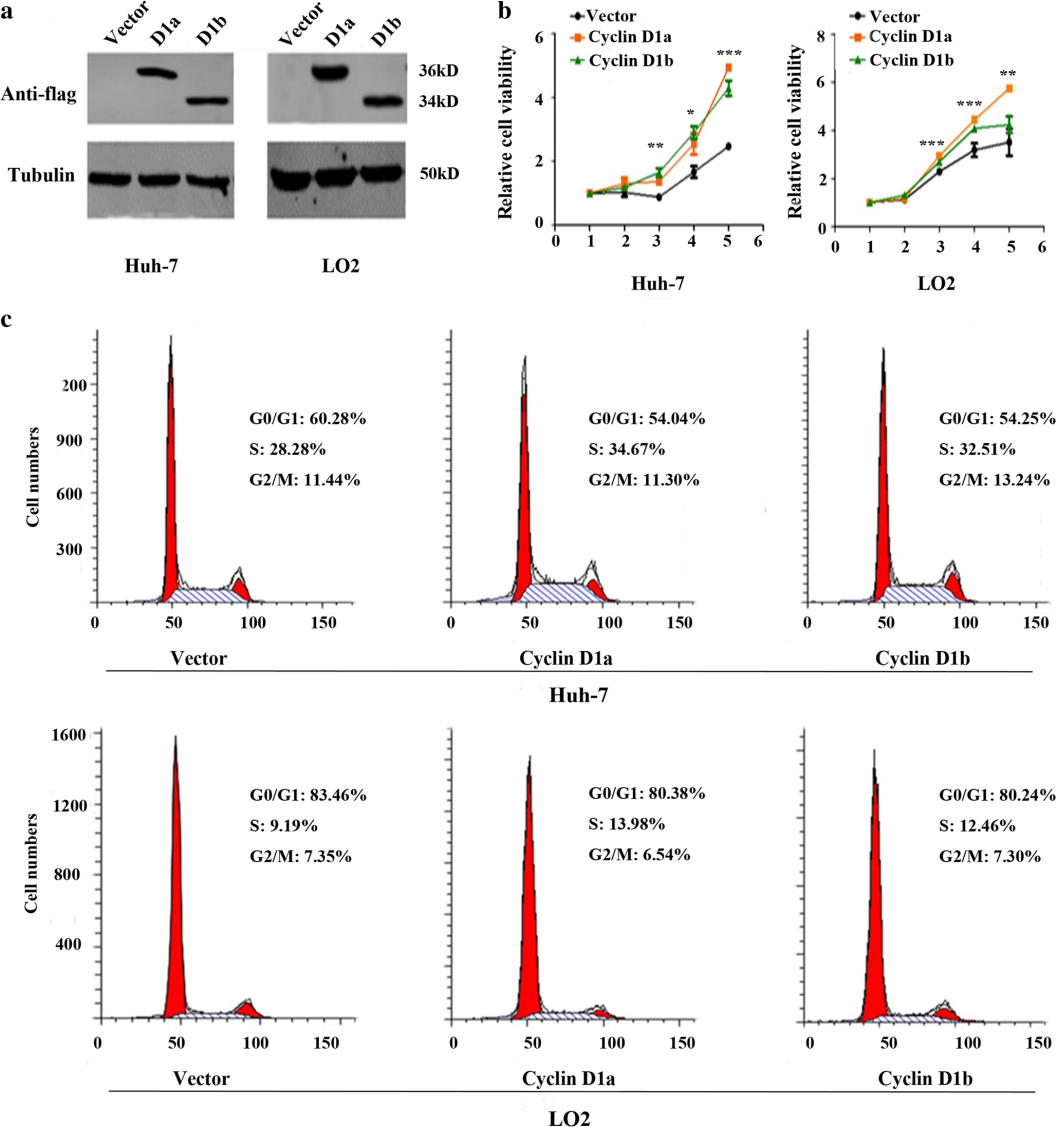
Epitopic expression of cyclin D1a and cyclin D1b promote the cell proliferation in Huh-7 and LO2 cells. Huh-7 and LO2 cells were transiently transfected with pFlex-cyclin D1a, pFlex-cyclin D1b, and pFlex vector. Ectopic expression of cyclin D1 variants was detected by Western blot analysis using Flag-tagged antibody (**a**). **b** The CCK-8 assay was performed to measure the growth curve of Huh-7 and LO2 cells transfected with plasmids as (**a**). All measurements were done in triplicate and data are shown as the means ± SD. *P* values < 0.05 (*), <0.01 (**) or <0.001 (***). **c** Cell cycle was detected by flow cytometry analysis in Huh-7 and LO2 cells transfected with plasmids as (**a**)

To investigate whether both cyclins D1a and D1b could affect the cell proliferation, we transiently transfected the cyclins D1a and D1b expression plasmids into HCC cell line Huh-7 and normal hepatic cell LO2. Western blot assay demonstrated the ectopic expression of Flag-tagged cyclins D1a and D1b protein in these cells (Fig. 4a). We then performed a CCK-8 cell proliferation assay to access the roles of both variants in cell growth. As shown in Fig. 4b, overexpression of both cyclins D1a and D1b could obviously promote cell proliferation in Huh-7 and LO2 cell lines as compared to their indicated vector control. Next, we measured the effects of cyclins D1a and D1b on the cell-cycle distribution. Consistent with the increased cell growth, both variants could reduce the cells in G0/G1 phase but increase cells in S phase (Fig. 4c). These results suggested that both cyclins D1a and D1b could promote cell proliferation via accelerating the cell-cycle progression. However, in both Huh-7 and LO2 cells, no difference of cell proliferation and cell-cycle distribution was found between the cyclins D1a and D1b transfected cells.

## Discussion

Although a number of studies have described that the *CCND1* 870A allele is associated with susceptibility to various tumors [7–10], results of recent meta-analysis indicated that *CCND1* G870A polymorphism is a potential risk factor only in the development of brain, lung, breast, and colorectal cancers [10, 19–22]. These results suggested that G870A polymorphism might contribute to the increased risk in different tumor type or ethnicity population. According to our recent knowledge, only three studies have been conducted to examine the associations between the G870A polymorphism and HCC risks [8, 23, 24]. The control subjects used in these studies were all recruited from the health examination individuals without evidence of HBV infection. Since most HCC patients in China have HBV infection and hepatic cirrhosis, it is imperative to explore association between G870A polymorphism and the risk of HBV-related HCC by using chronic HBV infection or cirrhotic CHB patients as control population.

In this study, we investigated the HCC tumor relevance of G870A polymorphism in a relatively large cohort, with the healthy control, CHB patients or cirrhotic CHB patients as the control population. We found that the G870A genotype had no association with the susceptibility of HBV-related HCC in this Chinese cohort. This result is consistent with two studies performed in a Taiwan or mainland Chinese population by Zhang et al. [23] and Hu et al. [24], which showed no association between G870A genotype and HCC risk in a Taiwan or mainland Chinese population by using healthy population as control. By contrast, the report of Akkiz et al. [8] identified a significant association between the A allele and the increased HCC risk in a Turkish population. Except the ethnic difference, the specific etiological difference of HCC may provide an alternative explanation of the above contradiction. Since chronic HBV infection is a well-known strong risk factor for HCC, it is reasonable to assume that its strong association with HCC may muffle the weak association of G870A polymorphism. Indeed, our stratification analysis by age revealed a weak association between AA genotype and higher HCC risk in younger cirrhotic CHB patients (ages ≤ 50). Similar to our results, Hu et al. [24] also found that the AG and AA genotypes were associated with an increased risk of HCC among HBsAg-positive individuals. However, larger well-designed studies are still required to evaluate the interaction of G870A polymorphism with HCC risk.

*CCND1* G870A polymorphism was thought to affect cyclin D1 variant expression in nonsmall cell lung cancer, head and neck squamous cell carcinoma, and prostate cancer [20, 25, 26]. In our study, we found that both A and G alleles can splice to form cyclin D1a or D1b, and the expression level of cyclin D1a or cyclin D1b had no association with the G870A genotype in HCC tumor, adjacent nontumor, and normal liver tissues. Our findings is consistent with the results from lymphoma and esophageal cancers [13, 27], supporting the notion that the alternative splicing of *CCND1* gene also depends on cell origin. It is interesting that both cyclin D1a and cyclin D1b transcripts had more G allele than A allele in AG heterozygous liver cells. One explanation is that G allele may have higher transcript efficiency than A allele in liver cells.

Although cyclin D1 overexpression shows a close association with malignancy, little is known about the status of cyclin D1a and cyclin D1b expression in HBV-related HCC. We found that the expression of both cyclins D1a and D1b are significantly lower in the tumor-derived specimens of HBV-related HCC as compared with the adjacent nontumor tissues. This result is consistent with numbers of research which also demonstrated that cyclin D1 was decreased in HCC samples [28–30], while inconsistent with a recent study which showed the overexpression of cyclin D1 in about 40 % of HCC samples [31]. Since these studies did not distinguish whether the HCC tissues have the background of HBV infection and liver cirrhosis, the conflicting results may be due to the complicated etiological mechanism of HCC. Interestingly, we also found that the cyclin D1 expression was significantly increased in HCC tumor tissues without HBV infection (Fig. S1), supporting the notion that the expression and function of cyclin D1 in HCC without HBV infection was different to that with HBV infection. At present, the mechanism underlying the lower levels of cyclin D1 expression in HBV-related HCC tissues is unknown. Previous studies have reported that the viral proteins of HBV, such as HBx and HBsAg, could upregulate cyclin D1 expression through activating Wnt, Erk, and NF-κB pathways [32–34]. Moreover, studies on viral protein expression in HCC showed that the HBx or HBsAg expression level was higher in nontumor tissues as compared with that in tumor tissues [33, 35]. Based on these findings, we assumed that the downregulation of cyclin D1 in HBV-related HCC tissues may be linked to the reduced HBx or HBsAg expression. Further study should be performed to investigate the specific expression pattern of each cyclin D1 variant and the underlying mechanism of its downregulation in the HBV-related HCC tissues.

In the case of HBV-related HCC, we noticed that the expression of both cyclins D1a and D1b was increased in adjacent nontumor cirrhotic tissues as compared with that in normal liver tissues. It has been reported that the cirrhosis regenerative nodules may progress along a well-described carcinogenic pathway to become dysplastic nodules or hepatocellular carcinomas [36]. Since our study have demonstrated that overexpression of both cyclin D1a and cyclin D1b could promote cell proliferation by accelerating the cell cycle, it is reasonable to suppose a potential oncogenic role for both D1 variants during the progression of HBV-associated hepatocarcinogenesis from as early as the precancerous cirrhotic stage. Then the next question to consider is that how HCC tumor cells remain their uncontrolled proliferation without cyclin D1 overexpression in HCC. It is known that cyclin D1 promotes cell-cycle progression via binding to and activation of cyclin-dependent kinases 4 and 6 (CDK4/6). The cyclin D1/CDK complexes phosphorylate and inactivate the retinoblastoma protein (Rb), thereby triggering E2F-dependent transcription of genes required for S-phase entry [37]. Therefore, the loss of the functional pRB also could keep driving cell-cycle progression and proliferation independently of the high cyclin D1 level. Indeed, other studies and our previous aCGH data have identified a frequently functional loss of RB gene in HBV-related HCC [38, 39].

Moreover, besides G870A polymorphism, it is now clear from a number of studies that other factors, such as chromatin-remodeling protein Brm, RNA-binding protein Sam68, and Ewing’s sarcoma oncogene, may also modulate the expression of cyclin D1b [40–42]. Thus, the relative production of cyclin D1b and cyclin D1a may reflect differences in the tumors’ specific signaling pathways. However, we found that the cyclin D1b/D1a ratio in HCC tumor tissues showed no difference with that in adjacent nontumor tissues and normal liver tissues. This finding is supported by our analyses of linear relationship between cyclins D1a and D1b expression in both tumor and nontumor tissues. Since the cyclins D1a and D1b are transcribed from the same gene, these data indicated that the expression patterns of cyclin D1b are similar to that of cyclin D1a in liver tissues. Thus, the same mechanism underlying the regulation of both isoforms may exist in liver tissues.

In summary, the data herein demonstrated that the G870A polymorphism is not a strong predictor of the HBV-related HCC risk in Chinese population. The two variants were both transcripted from A and G alleles, and neither cyclin D1a nor cyclin D1b production was influenced by G870A genotype in HCC. Both cyclins D1a and D1b could promote cell proliferation, which might contribute to the potential oncogenic role of cyclin D1 variants during the precancerous cirrhotic stage of HBV-associated hepatocarcinogenesis.

## Electronic supplementary material

Below is the link to the electronic supplementary material.

ESM 1 (DOCX 28 kb)

ESM 2 (DOCX 753 kb)
