# Supplementary material for: Influence of CCND1 G870A polymorphism on the risk of HBV-related HCC and cyclin D1 splicing variant expression in Chinese population
Source: Tumour Biol. 2015 Apr 8;36(9):6891–900. doi: 10.1007/s13277-015-3401-7 (PMC4644212; doi:10.1007/s13277-015-3401-7)
Supplement: Supplementary file 1 — (DOCX 28 kb) [file 13277_2015_3401_MOESM1_ESM.docx]

**Supplementary Table 1. Associations and stratification analysis of cyclin D1 G870A polymorphism and HCC risk**

|  | **AA (%)** | **AG (%)** | | **GG (%)** | ***p-value****^a^* | **OR (95% CI)^b^** | **A (%)** | **G (%)** | ***p-value****^a^* | **OR (95% CI)** |
| --- | --- | --- | --- | --- | --- | --- | --- | --- | --- | --- |
| ***Gender*** |  |  | |  |  |  |  |  |  |  |
| **male** |  |  | |  |  |  |  |  |  |  |
| CHB | 54(31) | 79(45.4) | | 40(23.6) | *0.7805* | 0.748[0.417,1.34] | 187(54.05) | 159(45.95) | *0.777* | 0.959[0.72,1.278] |
| cirrhotic CHB | 39(22.8) | 88(51.5) | | 44(25.7) | *0.0713* | 0.794[0.473,1.333] | 166(48.54) | 176(51.46) | *0.073* | 0.769[0.577,1.025] |
| HCC | 62(29.95) | 104(50.24) | | 41(19.81) | *─* | *─* | 228(55.07) | 186(44.93) | *─* | ─ |
| **female** |  |  | |  |  |  |  |  |  |  |
| CHB | 13(18.8) | 38(55.1) | | 18(26.1) | *0.7209* | 1.161[0.224,6.016] | 64(46.37) | 74(53.62) | *0.559* | 1.198[0.654,2.194] |
| cirrhotic CHB | 14(21.5) | 35(53.8) | | 16(24.6) | *0.3822* | 1.158[0.428,5.901] | 63(48.46) | 67(51.54) | *0.396* | 1.302[0.707,2.397] |
| HCC | 5(16.13) | 16(51.61) | | 10(32.26) | *─* | *─* | 26(41.94) | 36(58.06) | *─* | ─ |
| ***Age*** |  |  | |  |  |  |  |  |  |  |
| **≤50** |  |  | |  |  |  |  |  |  |  |
| CHB | 56(28.5) | 93(46.7) | | 50(25.1) | *0.2756* | 0.821[0533,1.264] | 205(51.51) | 193(48.49) | *0.262* | 0.811[0.562,1.169] |
| cirrhotic CHB | 24(20.2) | 64(53.8) | | 31(26.1) | ***0.0547*** | **1.052[1.000,1.106]** | 112(47.06) | 126(52.94) | ***0.057*** | **0.679[0.455,1.013]** |
| HCC | 27(32.9) | 39(47.6) | | 16(19.5) | *─* | *─* | 93(556.71) | 71(43.29) | *─* | ─ |
| **>50** |  |  | |  |  |  |  |  |  |  |
| CHB | 11(25) | 24(54.5) | | 9(20.5) | *0.9094* | 1.086[0.655,1.801] | 46(52.27) | 42(42.73) | *0.912* | 1.027[0.64,1.649] |
| cirrhotic CHB | 29(24.8) | 59(50.4) | | 29(24.8) | *0.7077* | 1.047[0.731,1.502] | 117(50) | 117(50) | *0.711* | 0.938[0.668,1.316] |
| HCC | 40(25.8) | 81(51.6) | | 35(22.6) | *─* | *─* | 161(51.60) | 151(48.40) | *─* | ─ |
| ***Serum HBV DNA level(IU/ml)*** | | | |  |  |  |  |  |  |  |
| **HBV DNA≤10^5^** |  | |  |  |  |  |  |  |  |  |
| CHB | 24(32.4) | | 31(41.9) | 19(25.7) | *0.381* | 1.043[0.414,2.629] | 79(53.38) | 69(46.62) | *0.958* | 0.989[0.667,1.468] |
|  | **AA (%)** | | **AG (%)** | **GG (%)** | ***p-value****^a^* | **OR (95% CI)^b^** | **A (%)** | **G (%)** | ***p-value****^a^* | **OR (95% CI)** |
| cirrhotic CHB | 27(27) | | 53(53) | 20(20) | *0.9784* | 0.841[0.364,1.941] | 107(53.50) | 93(46.50) | *0.975* | 0.994[0.695,1.423] |
| HCC | 42(27.8) | | 78(51.7) | 31(20.5) | *─* | *─* | 162(53.64) | 140(46.36) | *─* | ─ |
| **HBV DNA>10^5^** |  | |  |  |  |  |  |  |  |  |
| CHB | 43(25.6) | | 86(51.2) | 39(23.2) | *0.8393* | 0.888[0.382,2.063] | 172(51.19) | 164(48.81) | *0.624* | 0.912[0.631,1.318] |
| cirrhotic CHB | 26(19.1) | | 70(51.5) | 40(29.4) | *0.1822* | 0.525[0.238,1.154] | 122(44.85) | 150(55.15) | *0.076* | 0.707[0.482,1.038] |
| HCC | 25(29.1) | | 42(48.8) | 19(22.1) | *─* | *─* | 92(53.49) | 80(46.51) | *─* | ─ |
| ***HBV genotype*** | | | |  |  |  |  |  |  |  |
| **B** | | | |  |  |  |  |  |  |  |
| CHB | 10(19.6) | | 29(56.9) | 12(23.5) | *0.3588* | 2.069[0.211,20.27] | 49(48.04) | 53(51.96) | *0.716* | 1.233[0.399,3.807] |
| cirrhotic CHB | 3(18.8) | | 9(56.2) | 4(25) | *0.4709* | 1.296[0.11,15.254] | 15(46.87) | 17(53.13) | *0.801* | 1.176[0.332,4.172] |
| HCC | 2(28.6) | | 2(28.6) | 3(42.8) | *─* | *─* | 6(42.86) | 8(57.14) | *─* | ─ |
| **C** | | |  |  |  |  |  |  |  |  |
| CHB | 43(28.5) | | 73(48.3) | 35(23.2) | *0.3988* | 0.895[0.44,1.821] | 159(52.65) | 143(47.35) | *0.52* | 0.907[0.674,1.221] |
| cirrhotic CHB | 37(22.3) | | 90(54.2) | 39(23.5) | *0.9795* | 0.769[0.414,1.427] | 164(49.4) | 168(50.6) | *0.123* | 0.796[0.596,1.064] |
| HCC | 58(28.7) | | 102(50.5) | 42(20.8) | *─* | *─* | 228(55.07) | 186(44.93) | *─* | ─ |
| ***ALT(U/L)*** |  | |  |  |  |  |  |  |  |  |
| **≤40** |  | |  |  |  |  |  |  |  |  |
| CHB | 6(31.6) | | 9(47.4) | 4(21.) | *0.8114* | 2.281[0.456,11.424] | 21(55.26) | 17(44.74) | *0.777* | 1.107[0.548,2.234] |
| cirrhotic CHB | 17(20) | | 44(51.8) | 24(28.2) | *0.3793* | 0.77[0.3,1.977] | 78(45.88) | 92(54.12) | *0.198* | 0.76[0.499,1.155] |
| HCC | 23(25.3) | | 50(54.9) | 18(19.8) | *─* | *─* | 96(52.75) | 86(47.25) | *─* | ─ |
| **>40** |  | |  |  |  |  |  |  |  |  |
| CHB | 61(27.2) | | 108(48.2) | 55(24.6) | *0.81* | 0.791[0.404,1.549] | 230(51.34) | 218(48.66) | *0.518* | 0.907[0.675,1.219] |
|  | **AA (%)** | | **AG (%)** | **GG (%)** | ***p-value****^a^* | **OR (95% CI)^b^** | **A (%)** | **G (%)** | ***p-value****^a^* | **OR (95% CI)** |
| cirrhotic CHB | 35(23.3) | | 79(52.7) | 36(24) | *0.4111* | 0.762[0.395,1.469] | 149(49.67) | 151(50.33) | *0.318* | 0.848[0.614,1.171] |
| HCC | 44(30.1) | | 69(47.3) | 33(22.6) | *─* | *─* | 157(53.77) | 135(46.23) | *─* | ─ |
| ***AST(U/L)*** | | |  |  |  |  |  |  |  |  |
| **≤40** |  | |  |  |  |  |  |  |  |  |
| CHB | 9(36) | | 12(48) | 4(16) | *0.5704* | 0.632[0.135,2.971] | 30(60) | 20(40) | *0.64* | 1.171[0.603,2.274] |
| cirrhotic CHB | 10(23.3) | | 21(48.8) | 12(27.9) | *0.1922* | 0.409[0.143,1.167] | 41(47.67) | 45(52.33) | *0.222* | 0.711[0.412,1.229] |
| HCC | 17(26.2) | | 39(60) | 9(13.8) | *─* | *─* | 73(56.15) | 57(43.85) | *─* | ─ |
| **>40** |  | |  |  |  |  |  |  |  |  |
| CHB | 58(26.6) | | 105(48.2) | 55(25.2) | *0.8643* | 1.433[0.302,6.8] | 221(50.69) | 215(49.31) | *0.65* | 0.937[0.706,1.243] |
| cirrhotic CHB | 42(21.9) | | 102(53.1) | 48(25) | *0.2639* | 0.613[0.175,2.147] | 186(48.44) | 198(51.56) | *0.295* | 0.856[0.64,1.145] |
| HCC | 50(29.1) | | 80(46.5) | 42(24.4) | *─* | *─* | 180(52.33) | 164(47.67) | *─* | ─ |

*^a^* Pearson χ^2^ –test

^b^ Adjusted for age and sex by logistic regression analysis. GG and GA genotypes were used as reference.

Note: CHB, chronic hepatitis B virus infection; HCC, hepatocellular carcinoma.
