# Supplementary material for: Influence of CCND1 G870A polymorphism on the risk of HBV-related HCC and cyclin D1 splicing variant expression in Chinese population
Source: Tumour Biol. 2015 Apr 8;36(9):6891–900. doi: 10.1007/s13277-015-3401-7 (PMC4644212; doi:10.1007/s13277-015-3401-7)
Supplement: Supplementary file 2 — (DOCX 753 kb) [file 13277_2015_3401_MOESM2_ESM.docx]

**Supplementary Figure**

**
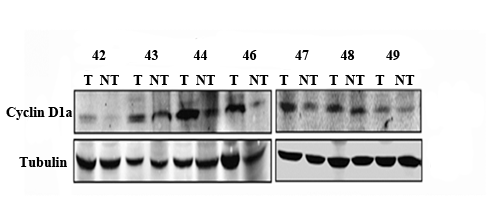
**

**Fig.S1** **Expression of cyclin D1 in paired HCC tissues without HBV infection.** Western blot assay was performed on 7 paired HCC tissues and the corresponding non-tumor tissues to detect the expression of cyclin D1 protein, all the 7 paired tissues were without HBV infection. Each sample was tested in triplicate. T, tumor tissue; NT, non-tumor tissue.
